# The anti-diabetic effects of metformin are mediated by regulating long non-coding RNA

**DOI:** 10.3389/fphar.2023.1256705

**Published:** 2023-11-20

**Authors:** Wenguang Chang, Wei Li, Peifeng Li

**Affiliations:** ^1^ Institute for Translational Medicine, The Affiliated Hospital, College of Medicine, Qingdao University, Qingdao, China; ^2^ College of Chinese Medicinal Materials, Jilin Agricultural University, Changchun, China

**Keywords:** long non-coding RNA, T2DM, metformin, mechanism, AMPK

## Abstract

Type 2 diabetes (T2D) is a metabolic disease with complex etiology and mechanisms. Long non-coding ribonucleic acid (LncRNA) is a novel class of functional long RNA molecules that regulate multiple biological functions through various mechanisms. Studies in the past decade have shown that lncRNAs may play an important role in regulating insulin resistance and the progression of T2D. As a widely used biguanide drug, metformin has been used for glucose lowering effects in clinical practice for more than 60 years. For diabetic therapy, metformin reduces glucose absorption from the intestines, lowers hepatic gluconeogenesis, reduces inflammation, and improves insulin sensitivity. However, despite being widely used as the first-line oral antidiabetic drug, its mechanism of action remains largely elusive. Currently, an increasing number of studies have demonstrated that the anti-diabetic effects of metformin were mediated by the regulation of lncRNAs. Metformin-regulated lncRNAs have been shown to participate in the inhibition of gluconeogenesis, regulation of lipid metabolism, and be anti-inflammatory. Thus, this review focuses on the mechanisms of action of metformin in regulating lncRNAs in diabetes, including pathways altered by metformin via targeting lncRNAs, and the potential targets of metformin through modulation of lncRNAs. Knowledge of the mechanisms of lncRNA modulation by metformin in diabetes will aid the development of new therapeutic drugs for T2D in the future.

## Introduction

Type 2 diabetes (T2DM) is a disease that is characterized by chronic high blood glucose and/or insulin resistance in multiple organs. If lifestyle changes and exercise in patients of T2DM cannot achieve satisfactory results, oral hypoglycemic drugs are introduced (Ganesan et al., 2023). Current treatment guidelines recommend the use of metformin as first-line therapy for T2DM (Lv and Guo, 2020). Metformin has been shown to lower fasting plasma glucose and HbA1c in a dose-related manner in patients with T2DM (Garber et al., 1997) and is associated with reduced body weight and waist circumference (Diabetes Prevention Program Research, 2012). Furthermore, although controversial, studies from the United Kingdom Prospective Diabetes Study and the Hyperinsulinaemia the Outcome of its Metabolic Effects (HOME) trial have shown that metformin has favorable effects on cardiovascular outcomes in both new-onset and advanced type 2 diabetes (Effect of intensive blood, 1998; Jager et al., 2005), as well as in kidney disease in patients with T2DM (Charytan et al., 2019the other hand, metformin inhibits mitochondrial). However, the molecular mechanisms of metformin remain largely elusive. In this paper, we highlight the mechanisms of metformin in current studies and focus on the action of metformin in regulating lncRNAs in T2DM. Exploring the action mechanisms of metformin will help to better guide clinical medication and find new drug targets for T2DM therapy.

## Molecular pathway for the anti-diabetic effects of metformin

### AMPK-dependent pathway

**FIGURE 1:**
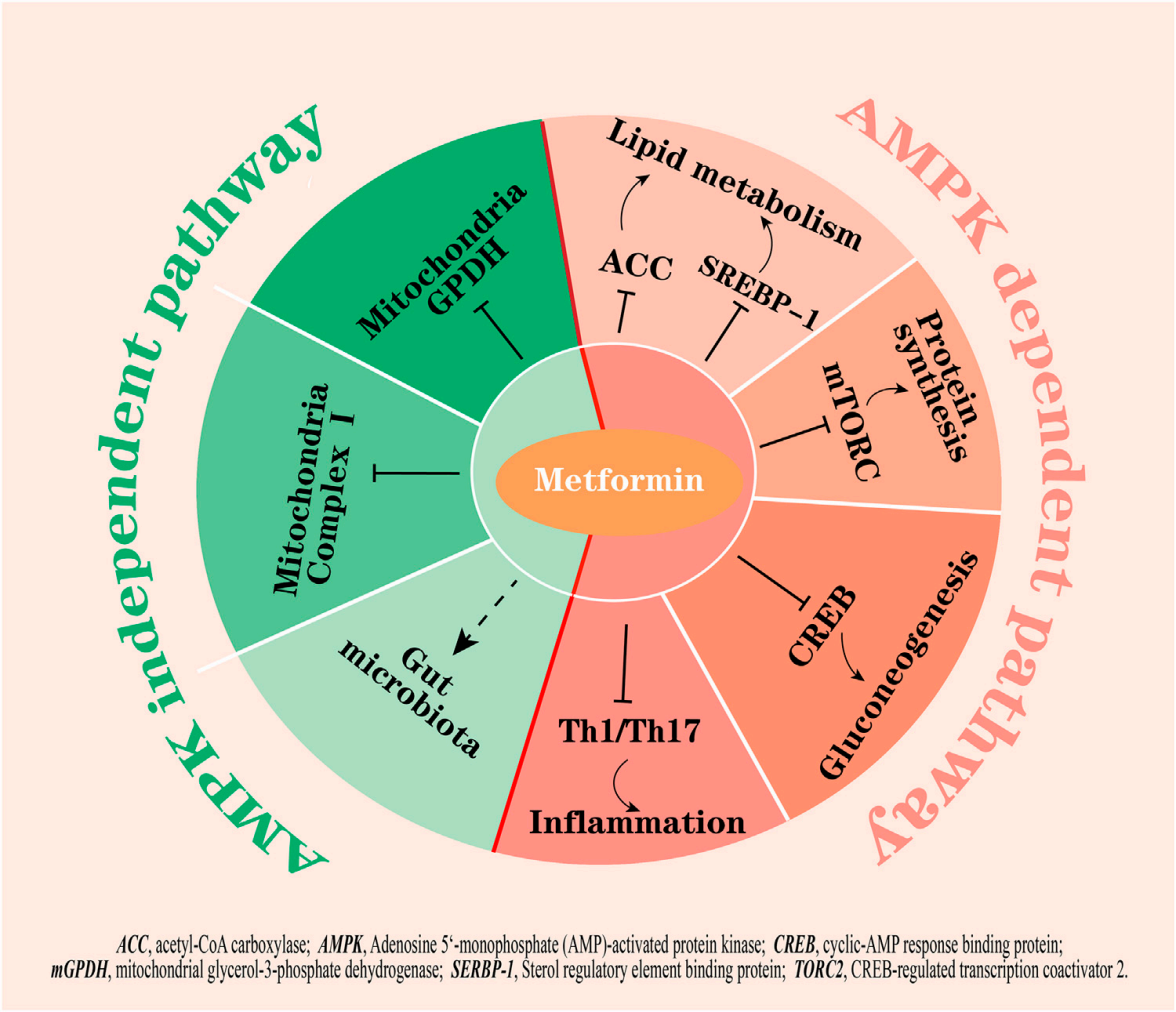
The hypoglycemic mechanisms of metformin by AMPK dependent and AMPK independent pathways. Activation of AMPK by metformin: 1) inhibits fatty acid synthesis by suppressing ACC and SREBP-1; 2) inhibits mTORC and CREB to inhibit protein synthesis and gluconeogenesis; and 3) inhibits the differentiation of Th1 and Th17 cells to exert anti-inflammation effects. On the other hand, metformin inhibits mitochondrial complex I and mGPDH, potentially modulates gut microbiota, and is not dependent on AMPK activation. Right part of red line, AMPK dependent pathways; left part of red line, AMPK independent pathways.

The molecular targets of metformin have been expanded upon in the recent decades (Foretz et al., 2019; Lu et al., 2021; Wu et al., 2023) but the primary mechanism of this biguanide remains unclear. The activation of adenosine 5’-monophosphate activated protein kinase (AMPK) is considered a core mechanism of metformin; AMPK phosphorylates several key enzymes that regulate the activity of transcription factors, coactivators, and corepressors, thus regulating lipid metabolism, inflammation, autophagy, and gluconeogenesis (Wang et al., 2019a; Yang et al., 2019; Zhang et al., 2022a; Li et al., 2022; Yang et al., 2022) (Figure 1). For example, AMPK activation suppresses the expression of a key lipogenic transcription factor, SREBP-1 in the liver, and activates AMPK phosphorylated acetyl-CoA carboxylase (ACC), which is an inhibitory phenotype and suppresses malonyl-CoA biosynthesis, thus inhibiting fatty acid synthesis and increasing fatty acid oxidation (Zhou et al., 2001). Furthermore, activated AMPK inhibits the activity of the mammalian target of rapamycin complex (mTORC) to induce cellular autophagy (Wang et al., 2021a) and decrease protein synthesis (Knudsen et al., 2020). The inhibition of fatty acids and protein synthesis can promote cellular glucose uptake and metabolism to lower glucose levels (Lundsgaard et al., 2020). On the other hand, metformin-induced activation of AMPK has been shown to phosphorylate CREB-regulated transcription coactivator 2 (CRTC2, also known as TORC2), which prevents its translocation to the nucleus, thus inhibiting the transcriptional activation of gluconeogenic genes (Koo et al., 2005). Moreover, metformin inhibits Th1 and Th17 cells differentiation while promoting Tregs development through activation of AMPK (Duan et al., 2019), which may contribute to its anti-inflammatory effects.

### AMPK-independent pathway

However, many studies showed that the beneficial effects of metformin remained in a transgenic mouse model with lacked AMPK or its upstream activator LKB1 (Foretz et al., 2010), indicating that there are other critical targets other than AMPK for metformin action. In fact, increasing evidence points to other actions of metformin, for example, metformin has been shown to inhibit mitochondrial complex Ⅰ, which is independent of AMPK activity in cells (El-Mir et al., 2000; Owen et al., 2000); the inhibition results in a decrease in ATP production, thus suppressing gluconeogenesis in the liver. Additionally, metformin at low doses (<130 µM in plasma) was reported to inhibit mitochondrial glycerol-3-phosphate dehydrogenase (mGPDH) activity, resulting in an increased hepatic cytosolic NADH to NAD^+^ ratio (lactate to pyruvate ratio) independently of changes in intracellular levels of ATP and inhibition of glucose production from reduced gluconeogenic substrates (lactate and glycerol) (Madiraju et al., 2014; Madiraju et al., 2018). In addition to these, a study found that glucose tolerance improved in germ-free mice that received metformin-altered microbiota via fecal samples transfer, indicating that the altered gut microbiota mediates some of the antidiabetic effects of metformin (Wu et al., 2017) (Figure 1).

## LncRNA biogenesis and expressions in T2DM

Long non-coding RNAs (LncRNAs) are non-coding RNA transcripts that are more than 200 nucleotides (nt) in length. According to their location in the gene relative to nearby protein-coding genes, lncRNAs are classified as intronic lncRNAs, antisense lncRNAs, and long “intergenic” non-coding RNAs (lincRNAs). The biogenesis of lncRNAs is similar to that of mRNAs, but lncRNAs have some unique features that can distinguish them from mRNAs, for example, lncRNAs have relatively low expression and the presence of fewer and longer exons than mRNAs (Liu et al., 2021; Liu et al., 2023). Although they cannot produce proteins, they can act as pathway switches to participate in the regulation of signaling pathways. LncRNAs can function as scaffolds (bringing two distant genes closer), decoys (binding to transcription factors to inhibit gene transcription), guiders (guiding regulatory proteins to gene sequences), and genomic targeting (directly binding to gene sequences to affect gene expression), thus participating in molecular and genomic modulation (Panni et al., 2020). Meanwhile, a single lncRNA may have multiple targets, so its function may be diverse; additionally, lncRNAs are less species conserved compared to miRNAs, which makes the investigation of lncRNAs difficult.

Various pathological processes of diseases are mediated by lncRNAs (Liu et al., 2022a; Zhou et al., 2022; Ao et al., 2023; Mattick et al., 2023). Several lncRNAs function as biomarkers or intervention targets that aid in the diagnosis and treatment of T2DM. For example, abnormal expressions of lncRNA, including CASC2, GAS5, ENST00000550337.1, HOTAIR, MEG3, H19, MALAT1, MIAT, ANRIL, XIST, PANDA, Linc-p21, PLUTO, and NBR2 in blood samples are closely associated with diagnostic outcomes of T2DM (Sathishkumar et al., 2018). Furthermore, some lncRNAs participate in the progression of diabetic complications, such as plasma taurine upregulated gene 1 (TUG1) which is associated with the progression of diabetic nephropathy in diabetic rats and Kcnq1ot1 which is involved in the progression of diabetic cardiomyopathy (Yang et al., 2018a). In addition, β cell replacement is the other way to treat diabetes; in recent years, 2D and 3D models based on induced pluripotent stem cells (iPSCs) have attracted much attention to provide a new approach to β-like cell replacement therapies, and lncRNAs play an essential role in the regulatory mechanisms of cell reprogramming and differentiation (Ilieva and Uchida, 2022). For example, the lncRNA Gm10451 is a potential regulator for the β-like cell differentiation of iPSCs (Huang et al., 2019).

## Metformin mediates lncRNA expressions

Metformin treatment can alter the expressions of lncRNAs. How metformin acts on lncRNAs is not clear; a bioinformatics study showed that metformin promotes different expressions of lncRNA isoforms among lung cells, kidney cells, embryonic cells, hepatocytes, and pancreatic cells. For example, NEAT1-202, the canonical isoform of the NEAT1 gene, is upregulated in five out of six cell lines by metformin treatment, but other isoforms showed different fold change directions (Conceicao et al., 2022). This may explain different results for the same lncRNA in different studies. In addition, the presence of upregulated DNA methylation was found during metformin treatment. In these studies, DNMT1, the principal DNA methyltransferase in mammalian cells, was found to be increased by metformin. Hypermethylation can occur in lncRNAs; metformin has been shown to decrease H19 levels by inducing H19 methylation, which has been reported in cancer cells (Yan et al., 2015) and in animal models of pre-eclampsia (Shu et al., 2019).

Furthermore, the effects of metformin on lncRNAs depend on cell types and organs. For example, Dreh has been shown to be downregulated by a high-fat diet (HFD), and metformin has been shown to upregulate Dreh and improve lipid accumulation in mice livers (Guo et al., 2018). However, in C2C12 skeletal muscle cells, metformin-repressed Dreh expression induces an increase in glucose uptake (Takahashi et al., 2019). Furthermore, metformin was shown to decrease H19 in rodent models of HFD-induced liver disease (Guo et al., 2018), gastric cancer (Li et al., 2019), endometrial cancer (Aminimoghaddam et al., 2021), and breast cancer (Chen et al., 2022). However, metformin co-treatment with sitagliptin was shown to increase H19 expression to attenuate apoptosis and insulin resistance in rats with polycystic ovary syndrome (POCS) (Wang et al., 2019b). Similarly, the metformin-regulated expression of MALAT1, HOTAIR, and TUG1 was reported to be upregulated in breast cancer cells (Huang et al., 2021). However, other studies reported a decrease in the expression of TUG1 by metformin in vascular wall cells (You et al., 2020), a decrease in the expression of HOTAIR by metformin in MDA-MB-231 breast cancer cells (Golshan et al., 2021), and a decrease in the expression of MALAT1 by metformin in cervical cancer cells (Xia et al., 2018). The differences in metformin regulation suggest a necessity to differentiate the effects according to the state of the disease being treated. In diabetes conditions, metformin-induced alterations in lncRNA expression involve changes in gene transcription for glucose and lipid metabolism, the regulation of inflammatory cytokines, and the modulation of mitochondrial function.

## The anti-diabetic action of metformin mediated by lncRNAs

### Gluconeogenesis and glucose uptake

Inhibiting hepatic gluconeogenesis is the main mechanism of the blood glucose lowering action of metformin. However, the mechanisms underlying these effects are unclear. Studies have showed that the overexpression of the peroxisome proliferator-activated receptor-γ coactivator-1α (PGC-1α) preserved metformin’s ability to reduce glucose output regardless of AMPK expression (Foretz et al., 2010), suggesting that PGC-1α is a key factor for metformin’s gluconeogenic inhibitory effects. Concomitantly, another study found that cyclic adenosine monophosphate (cAMP), a gluconeogenic stimulus, induces changes in the expression of several lncRNAs in primary mouse hepatocytes. Furthermore, metformin treatment preserved 189 of the 456 upregulated lncRNAs and 167 of the 409 downregulated lncRNAs. Among those changed lncRNAs, NR_027710 and ENSMUST00000138573 were identified as having an association with the gene expression of PGC-1α (Wang et al., 2018), suggesting that metformin inhibiting gluconeogenesis could be mediated by the regulation of NR_027710 and ENSMUST00000138573. Consistent with these results, another study showed that 3,076 lncRNAs (1,823 upregulated and 1,253 downregulated) are altered in HFD-induced insulin resistant mice compared with the normal control group, and metformin treatment reversed 588 lncRNAs. Among those lncRNAs downregulated by metformin, NONMMUT031874.2 was highly expressed and found to target miR-7054-5p. Downregulating the expression of NONMMUT031874.2 by metformin could increase the expression of miR-7054-5p and then downregulate the expression of Socs3, which inhibits PEPCK to inhibit gluconeogenesis (Zhang et al., 2022b).

**FIGURE 2:**
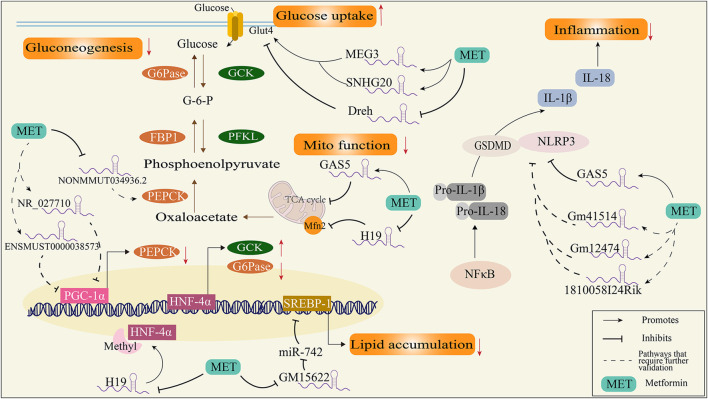
Schematic diagram of the mechanism of metformin regulating diabetes through lncRNAs. FBP1, fructose-1,6-bisphosphatase; G6Pase, glucose-6-phosphatase; GCK, glucokinase; GSDMD, gasdermin-D; HNF-4α, transcription factor hepatocyte nuclear factor 4α; IL-18, interleukin 18; IL-1β, interleukin 1β; Mfn2, mitofusion protein 2; NLRP-3, NOD-like receptor protein; NF-κB, nuclear factor-κB; PEPCK, phosphoenolpyruvate carboxylase kinase; PFKL, phosphofructokinase; PGC-1α, peroxisome proliferator-activated receptor-gamma coactivator; SERBP-1, sterol regulatory element binding protein.

In addition, hepatic gluconeogenesis is catalyzed by multiple enzymes, including G6Pase, PEPCK, and FBP1 (Mues et al., 2009). Suppressing hepatic gluconeogenesis is usually accompanied by the activation of glycolysis enzymes, including GCK, PFKL, and PK (Figure 2) (Al-Oanzi et al., 2017). Metformin treatment decreased the expression of lncRNA-NONMMUT034936.2 in liver tissue from HFD mice and exhibited similar expression patterns with G6P target genes, indicating that metformin may affect gluconeogenesis by regulating NONMMUT034936.2 (Shu et al., 2021). Furthermore, transcription factor hepatocyte nuclear factor 4α (HNF-4α) binding to promoters of G6Pase or GCK is required for GCK gene activation and insulin-mediated G6Pase gene repression (Hirota et al., 2008). Metformin-exposed mouse fetuses were shown to elevate H19 expression (Deng et al., 2017), a lncRNA reported to be related to T2DM and influence DNA methylation (Ding et al., 2012), which induces hypomethylation and the overexpression of HNF-4α, thus efficiently repressing hepatic gluconeogenesis (Figure 2).

On the other hand, the increased glucose uptake effects by metformin are mediated by lncRNAs. LncRNA-Dreh is reduced by metformin in C2C12 myotubes. Furthermore, knockdown of Dreh resulted in reduced glucose concentrations in the culture medium and increased levels of GLUT4 protein in the plasma membrane. The overexpression of Dreh attenuated the glucose-lowering effect of metformin in myotubes (Takahashi et al., 2019), suggesting the blood glucose lowering actions of metformin are mediated by Dreh in the skeletal muscle cells. Moreover, metformin treatment is reported to increase GLUT4 expression by inducing expression of MEG3 and SNHG20, thus improving insulin resistance in the endometrium of PCOS rats (Liu et al., 2022b), indicating the metformin regulation of lncRNAs in glucose uptake is not specific to a type of lncRNA.

### Lipid metabolism

Dyslipidemia is common among patients with T2DM (prevalence > 75%) (Athyros et al., 2018). Aberrations in lipid metabolism contribute to the progression of T2DM and the risk of diabetic complications. For example, excessive dietary lipid intake and enhanced lipogenesis can cause ectopic lipids to accumulate in adipose, pancreatic, vascular, and cardiac cells (Yang et al., 2018b), resulting in atherosclerosis and cardiomyopathy due to insulin resistance (Wolf et al., 2014). Metformin has been shown to inhibit lipogenesis by suppressing sterol regulatory element binding protein 1 (SREBP-1) (Madsen et al., 2015), which is a transcriptional factor for lipogenesis. In particular, the inhibition of SREBP-1 by metformin is reported to be mediated by lncRNA; a study found that lncRNA-Gm15622 was upregulated in the liver of obese mice fed a HFD and in liver cells treated with saturated fatty acid. Metformin suppresses lncRNA-Gm15622 both *in vivo* and *in vitro*, thus decreasing the expression of SREBP-1c and inhibiting lipid accumulation by sponge for SREBP-1 targeted microRNA (Ma et al., 2020). Additionally, MEG3 and SNHG20 have been shown to be downregulated in HFD-induced fatty liver mice, and metformin improves lipid accumulation by reversing the expressions of MEG3 and SNHG20 (Guo et al., 2018). As stated above, MEG3 and SNHG20 were shown to regulate glucose uptake, indicating that these two lncRNAs may mediate the cross-talk of lipid and glucose metabolism via metformin.

### Inflammation

Chronic inflammation is found in adipose tissues, pancreatic islets, and the liver (Lontchi-Yimagou et al., 2013), which contributes to insulin resistance and progression to T2DM and the long-term complications of diabetes (Berbudi et al., 2020; Gasmi et al., 2021). The nucleotide-binding oligomerization domain leucine-rich repeat and pyrin domain-containing protein 3 (NLRP3) inflammasome has emerged as a key mediator of pathological inflammation in diabetes (Esser et al., 2014). Firstly, inflammatory stimuli (such as TNFα, SFA, or LPS) induce NF-κB expression and result in the expression of pro-IL-1β and pro-IL-18; secondly, NLRP3 and GSDMD mediated the maturation and secretion of IL-1β and IL-18 after activation. Subsequently, cells secrete a large number of pro-inflammatory cytokines (e.g., IL-1β and IL-18) and induce a type of cell death called pyroptosis (Ding et al., 2019). The specific regulatory mechanisms of NLRP3 inflammasome activation remain unclear, and lncRNAs were shown to modulate NLRP3 activation in diabetes. For example, growth arrest-specific 5 (GAS5) overexpression suppressed NLRP3 inflammasome activation-mediated pyroptosis by regulating the miR-34b-3p/AHR axis in diabetic cardiomyocytes (Xu et al., 2020a). Although there is no direct evidence showing that metformin could regulate NLRP3 by regulating GAS5, clinical trials showed that taking metformin for 2 months was shown to increase GAS5 in patients with T2D (Parvar et al., 2023), indicating its anti-inflammatory role in the diabetic heart. Furthermore, metformin significantly alleviated NLRP3 and GSDMD expressions in diabetic periodontitis mice with a severe inflammation state, and in this study, lncRNA_1810058I24Rik, lncRNA_Gm12474, and lncRNA_Gm41514 were found to be co-expressed with NLRP3 and GSDMD (Zhou et al., 2020), which may potentially be regulated by metformin. However, a study conducted on the mouse glomerular membrane epithelial cell line SV40-MES-13 showed that metformin treatment efficiently inhibits elevation of H19 via high glucose in cells, with decreased levels of TNF-a, IL-6, and TGFβ-1, and overexpressed H19 abolishes the effects of metformin in SV40-MES-13 cells (Xu et al., 2020b), suggesting that the anti-inflammation effects of metformin are related to the regulation of lncRNAs.

### Mitochondria

Mitochondrial dysfunction is a key pathological factor for diabetes. Reduced oxidative phosphorylation and excessive ROS production have been found in patients with obesity and type 2 diabetes (Wada and Nakatsuka, 2016; Luc et al., 2019). In addition, mitochondrial dynamic changes, such as mitochondrial fusion and fission, have been considered to be closely related to insulin sensitivity (Watanabe et al., 2014; Rovira-Llopis et al., 2017). For example, mice with knockdown of liver-specific mitofusin 2 (Mfn-2, a key protein for mitochondrial fusion) exhibit hepatic insulin resistance and glucose intolerant (Sebastian et al., 2012). For a decade, mitochondria have been considered a classic target for the blood glucose lowering actions of metformin. A most recent study identified the possible binding sites for biguanides on mitochondrial complex I protein subunits (Bridges et al., 2023). Additionally, mGPDH and complex IV have also been considered targets of metformin in animal experiments (Madiraju et al., 2014; LaMoia et al., 2022), and more targets have been found in the research into the actions of metformin in mitochondria (Pecinova et al., 2019; MacDonald et al., 2021). LncRNAs regulated by metformin potentially affect mitochondrial function; clinical research on DNA methylation in plasma from metformin-treated patients with T2DM showed that 57 lncRNA-associated DNA methylation regions were found, which included the mitochondrial ATP synthase-coupling factor 6 (ATP5J) (Solomon et al., 2020), suggesting that metformin may regulate ATP5J through lncRNAs. In addition, metformin increases GAS5 expression in HepG2 cells and the plasma of patients with T2DM (Parvar et al., 2023), and elevated GAS5 decreases mitochondrial tricarboxylic acid flux by disrupting the metabolic enzyme tandem of the tricarboxylic acid cycle (Sang et al., 2021), providing another explanation for the mitochondrial inhibition effects of metformin. Moreover, metformin decreases the expression of H19, while H19 has been shown to downregulate the expression of Mfn-2, a key protein for mitochondrial fusion, in type 1 diabetic renal tissues (Sultan et al., 2021), and a decline in Mfn2 was observed in both type 1 and type 2 diabetic rodents (Gao et al., 2012; Veeranki et al., 2016), suggesting that metformin may regulate Mfn2 by decreasing H19.

## Limitations and future directions

On one hand, the doses of metformin used in experiments are critical for its molecular actions on tissues or organs. It is reported that exposing the liver to metformin concentrations of >1 mM is reported to be supra-pharmacologic and will likely result in non-clinically relevant effects of metformin, and the inhibition effects of complex I and activation of AMPK by metformin are reported to be the results of these supra-pharmacologic effects. Indeed, studies have shown that micromolar metformin concentrations suppress glucose production in primary mouse hepatocytes that may not be observed in clinical conditions. In our paper, we found the lncRNA studies related to the anti-diabetic effects of metformin were mostly conducted using an oral administration of 100–300 mg/kg/d in mice (Table 1), which achieves approximate plasma concentrations of 50–150 μM according to other animal studies (LaMoia and Shulman, 2021), similar to humans taking 1 g of metformin twice daily (35 μM), but in cell experiments, the dosage range of metformin was wide, ranging from in 10 μM to 2 mM mostly achieving maximum effectiveness at 1 mM (Table 1). However, the mechanism studies have mostly been proved in cell experiments; therefore, further investigations are needed to confirm the results. Furthermore, the understanding of the molecular mechanisms of metformin at low doses in the treatment of diabetes is expanding a lot, such as its impact on the gut microbiota, its targeting of the lysosomal PEN2–ATP6AP1 axis (Shin et al., 2020; Sugawara and Ogawa, 2023), and its immunomodulatory effects (Alrifai et al., 2019; Saito et al., 2020), but the evidence for lncRNA participation in these effects is lacking.

**TABLE 1:** Doses of metformin used in T2DM for lncRNA study.

Doses/Concentrations in media	Duration	Administration route	Animal/Cell model	Limitations	Ref.
***In vivo***					
250 mg/kg/d	8 weeks	Oral	Mice	No cell experiments or in-depth mechanistic studies	Shu et al. (2021)
3 mg/kg/d	5 weeks	Oral	Mice	No cell experiments or in-depth mechanistic studies	Guo et al. (2018)
150–300 mg/kg/d	9 weeks	Oral	Mice	The concentration of metformin used in cell experiments far exceeds the drug concentration that can be achieved in serum after oral administration of metformin	Ma et al. (2020)
250 mg/kg/d	18 days	Oral	Pregnant mice	The object of study was fetuses	Deng et al. (2017)
200 mg/kg/d	6 weeks	Oral	Mice	The concentration of metformin used in cell experiments far exceeds the drug concentration that can be achieved in serum after oral administration of metformin	Zhang et al. (2022b)
200 mg/kg/d	8 weeks	Oral	Mice	No cell experiments related to metformin	Zhou et al. (2020)
1 g/d	60 days	Oral	Human	No cell experiments or in-depth mechanistic studies	Parvar et al. (2023)
***In vitro***					
2 mM	24 h	Added in medium	Hepatocytes	The concentration of metformin used in cell experiments far exceeds the drug concentration that can be achieved in serum after oral administration of metformin	Ma et al. (2020)
2 mM	8 h	Added in medium	Hepatocytes	The concentration of metformin used in cell experiments far exceeds the drug concentration that can be achieved in serum after oral administration of metformin	Wang et al. (2018)
2.5–69 mM	Not provided	Added in medium	Hepatocytes (2.5 mM), embryonic stem cells (10–25 mM), and lung/kidney cells (32–69 mM)	The concentration of metformin used in cell experiments far exceeds the drug concentration that can be achieved in serum after oral administration of metformin	Conceicao et al. (2022)
10 µM–100 µM	Not provided	Added in medium	Mouse glomerular membrane epithelial cell lines	No *in vivo* experiments	Xu et al. (2020b)
0.01–1 mM	48 h	Added in medium	Hepatocytes	The object of study was fetuses	Deng et al. (2017)
0.1–2 mM	24 h	Added in medium	Hepatocytes	The concentration of metformin used in cell experiments far exceeds the drug concentration that can be achieved in serum after oral administration of metformin	Zhang et al. (2022b)
0.25–1 mM	48 h	Added in medium	C2C12 myotubes	No *in vivo* experiments	Takahashi et al. (2019)

On the other hand, even in the same organ/tissue, metformin acts on multiple lncRNAs, and further research is needed on the lncRNAs that play the main role. Furthermore, the mechanism of actions of lncRNAs are not fully understood, and whether the regulatory effect of metformin on lncRNAs is the result of metabolic regulation or the reason for metabolic changes still needs further research. Moreover, current research on the mechanisms of lncRNAs focuses mainly on their function as molecular sponges for miRNA, but metformin itself also plays a role in miRNA expression (Wang et al., 2021b; Ao et al., 2023). Therefore, the relationship between the beneficial effects of metformin on metabolism and non-coding RNAs needs further clarification. Additionally, many lncRNAs, such as H19 and GAS5, have been shown to mediate the anti-diabetic effects of metformin, so the development of drugs targeting these lncRNAs may be a future direction for new drug research. However, the specificity of drug regulation of lncRNAs and the avoidance of possible side effects are problems that need to be solved.

## Conclusion

In this review, we found that metformin regulates lncRNAs to inhibit gluconeogenesis, increase glucose uptake, decrease lipid deposition, and alleviate inflammation, as well as potentially target mitochondria, thus demonstrating that metformin exerts global anti-diabetic effects by targeting lncRNAs and that these effects involve multiple signal pathways. In addition, we found that metformin doses may cause differences in experimental and clinical effects. A unified dose or finding a reasonable explanation is a direction of further research into the mechanisms of action of metformin. Overall, regulation of lncRNAs is emerging as a supplementary mechanism for the anti-diabetic action of metformin; the understanding of the diversity of lncRNA modification by metformin will deepen our knowledge of its pharmaceutical effects and provide new insights for diabetic therapy.

## Figures and Tables

**Table:** 

**lncRNAs**
**ANRIL**	anti-sense orientation upstream of the ARF gene
**CASC2**	cancer susceptibility candidate 2
**DREH**	downregulated expression by HBx
**GAS5**	growth arrest-specific transcript 5
**H19**	H19 imprinted maternally expressed transcript
**HOTAIR**	HOX transcript antisense RNA
**Kcnq1ot1**	Kcnq1 overlapping transcript 1
**MALAT1**	metastasis-associated lung adenocarcinoma transcript 1
**MEG3**	maternally expressed gene 3
**MIAT**	myocardial infarction associated transcript
**NEAT1**	nuclear paraspeckle assembly transcript 1
**PANDA**	P21-associated non-coding RNA DNA damage-activated
**PLUTO**	PDX1 regulated transcription
**PDX1**	PDX1 locus upstream transcript
**NBR2**	neighbor of BRCA1 gene 2
**SNHG20**	small nucleolar RNA host gene 20
**TUG1**	taurine upregulated gene 1
**XIST**	X-inactive specific transcript
**Others**
**ACC**	acetyl-CoA carboxylase
**AMPK**	adenosine 5‘-monophosphate (AMP)-activated protein kinase
**ATP5J**	ATP synthase-coupling factor 6
**DNMT1**	DNA methyltransferase 1
**FBP1**	fructose-1,6-bisphosphatase
**G6Pase**	glucose-6-phosphatase
**GCK**	glucokinase
**GLUT4**	glucose transport 4
**GSDMD**	gasdermin-D
**mGPDH**	mitochondrial glycerol-3-phosphate dehydrogenase
**HFD**	high-fat diet
**HNF-4α**	transcription factor hepatocyte nuclear factor 4α
**LKB1**	liver kinase B1; Mfn2, mitofusion protein 2
**NLRP-3**	NOD-like receptor protein
**PEPCK**	phosphoenolpyruvate carboxylase kinase
**PEN2**	presenilin enhancer 2
**PFKL**	phosphofructokinase
**PGC-1α**	peroxisome proliferator-activated receptor-gamma coactivator
**PK**	pyruvate kinase
**POCS**	polycystic ovary syndrome
**SERBP-1**	sterol regulatory element binding protein
**Socs3**	suppressor of cytokine signaling 3
**T2DM**	type 2 diabetes mellitus
**TORC2**	CREB-regulated transcription coactivator 2
